# Long-Term Efficacy and Safety of a Novel Low-Dose Triple Single-Pill Combination for the Treatment of Hypertension

**DOI:** 10.5334/gh.1481

**Published:** 2025-10-31

**Authors:** Abdul Salam, H. Asita de Silva, Dike Ojji, A. P. de Silva, G. Galappatthy, P. Lakshman, T. Kumanan, G. Mayurathan, T. Pereira, M. Rahuman, G. Ranasinghe, L. Rasnayake, W. Uluwattage, G. R. Constantine, Thambyaiah Kandeepan, Mahmoud Umar Sani, Amit Kumar, Rashmi Pant, William C. Cushman, Gian Luca Di Tanna, Diederick Grobbee, Krzysztof Narkiewicz, Suzanne Oparil, Neil R. Poulter, Markus P. Schlaich, Aletta E. Schutte, Wilko Spiering, Bryan Williams, Jackson T. Wright, Chris Gianacas, Mathangi Shanthakumar, Xiaoqiu Liu, Ruth Freed, Paul K. Whelton, Anthony Rodgers

**Affiliations:** 1 The George Institute for Global Health, University of New South Wales, AU; 2 Prasanna School of Public Health, Manipal Academy of Higher Education, IN; 3 Clinical Trials Unit, Faculty of Medicine, University of Kelaniya, LK; 4 Department of Internal Medicine, Faculty of Clinical Sciences, University of Abuja, NG; 5 Department of Medicine, Faculty of Medicine, University of Kelaniya, LK; 6 Institute of Cardiology, National Hospital of Sri Lanka, LK; 7 Jaffna Teaching Hospital, LK; 8 Kandy National Hospital, LK; 9 Colombo South Teaching Hospital, LK; 10 Kurunegala Teaching Hospital, LK; 11 Karapitiya Teaching Hospital, LK; 12 National Hospital of Sri Lanka, Colombo, LK; 13 Jaffna Teaching Hospital, Jaffna, LK; 14 Bayero University Kano & Aminu Kano, Teaching Hospital, Kano, NG; 15 The George Institute for Global Health, IN; 16 University of Tennessee Health Science Center, Tennessee, US; 17 University of Applied Sciences and Arts of Southern Switzerland, CH; 18 Julius Global Health, the Julius Center for Health Sciences and Primary Care, University Medical Center Utrecht, Utrecht University, NL; 19 Medical University of Gdańsk, PL; 20 University of Alabama at Birmingham, US; 21 Imperial College London, London, UK; 22 The University of Western Australia, AU; 23 University Medical Center Utrecht, Utrecht University, NL; 24 University College London, UK; 25 University Hospitals, Cleveland Medical Center, Case Western Reserve University, US; 26 Department of Epidemiology, Tulane University Celia Scott Weatherhead School of Public Health and Tropical Medicine, New Orleans, US

**Keywords:** hypertension, clinical trial, pharmacotherapy, global health

## Abstract

**Background:**

A novel low-dose triple single-pill combination of antihypertensive drugs (GMRx2) has demonstrated superior blood pressure (BP)-lowering efficacy compared to placebo and dual combinations in short-term randomized double-blind trials.

**Objectives:**

To evaluate the long-term BP-lowering efficacy and safety of GMRx2-based treatment when used in normal clinical care.

**Methods:**

After completing a four-week double-blind randomised phase, participants from Sri Lanka and Nigeria were enrolled into an open-label extension phase (OLE) with follow-up to one year. The OLE involved treatment and uptitration with GMRx2, of ¼, ½ and standard doses of telmisartan/amlodipine/indapamide (i.e., 10/1.25/0.625 mg, 20/2.5/1.25 mg and 40/5/2.5 mg), and add-on antihypertensive drugs if needed to target a home BP goal of <130/80 mm Hg. Home BP monitoring was continued throughout the follow-up and six follow-up clinic visits were conducted. The primary outcome was percentage of participants with home BP control (<130/80 mmHg) at week 52.

**Results:**

From 21 August 2023 to 20 August 2024, 50 participants participated in the OLE, of whom 48 (96%) completed it. The mean age of participants was 49 years and 60% were female. Home and clinic mean BP at enrolment into OLE were 126/79 mmHg and 131/83 mmHg, respectively. At one year, home BP control (<130/80 mmHg) was 60% and clinic BP control (<140/90 mmHg) was 88%. Home mean BP was reduced to 121/78 mmHg after 4 weeks into the OLE and was 120/78 mmHg at one year. For clinic BP, the corresponding values were 126/79 mmHg and 122/77 mmHg. None of the participants discontinued trial treatment due to an adverse event.

**Conclusions:**

In a population with mild-to-moderate hypertension, long-term therapy with GMRx2-based treatment achieved high levels of BP control and was well tolerated.

Trial registration: NCT04518306.

## Introduction

In randomized trials, initial or early treatment with low-dose triple single-pill combinations (SPCs) of antihypertensive drugs improved blood pressure (BP) control (1). However, such triple SPCs are currently unavailable in clinical practice. A novel low-dose triple SPC, GMRx2, has been developed in three strengths: at ¼, ½, and standard doses of telmisartan 10/20/40 mg, amlodipine 1.25/2.5/5 mg, and indapamide 0.625/1.25/2.5 mg, respectively (2). Two phase three randomized, double-blind trials have been completed to evaluate the efficacy and safety of the three GMRx2 strengths. The first trial showed superior efficacy and good tolerability of GMRx2 ¼ and GMRx2 ½ compared with placebo (3). The second trial showed superior efficacy and good tolerability of GMRx2 ½ and GMRx2 standard dose compared with three dual combinations of the component drugs at the same doses (4). Although the short-term efficacy and tolerability of GMRx2 have been evaluated, assessing its long-term efficacy and safety is crucially important, considering that hypertension is a chronic condition that requires prolonged treatment and sustained BP control to exert its full potential for prevention of cardiovascular disease (CVD) (5). Therefore, the purpose of this open-label extension phase (OLE) of a randomised trial was to evaluate the long-term efficacy and safety of a GMRx2-based treatment for BP lowering.

## Methods

### Design

This was a non-comparative, OLE following a randomized, double-blind, placebo-controlled phase of a multi-country trial (Supplementary Figure S1). The design and results of the randomized, double-blind phase have been previously published (2). Briefly, the trial included participants with mild-to-moderate BP elevation who were on no more than one antihypertensive drug, had a low risk for CVD, had no significant concomitant health conditions and no contraindications to the trial treatments, and both adhered to and tolerated a two-week placebo run-in phase after discontinuing any previous antihypertensive medication. Those with an average home systolic blood pressure (SBP) of 130–154 mmHg after the placebo run-in period were randomized to placebo, GMRx2 ¼, or GMRx2 ½ in a 1:2:2 ratio for four weeks. Overall, participants were randomized at 46 study sites located in five countries (Australia, Nigeria, Sri Lanka, the United States, and the United Kingdom) for the randomized, double-blinded phase. The OLE was conducted in two sites in Nigeria and nine sites in Sri Lanka.

### Participants

Participants were eligible to participate in the OLE provided they completed the randomized double-blind phase, had no contraindications to the GMRx2-based treatment, were willing to continue on GMRx2-based treatment until 52 weeks, and provided written, informed, consent.

### Intervention

After completing the randomized double-blind treatment at week 4, participants were immediately started on open label GMRx2 ¼. Clinic visits were initially conducted after two and four weeks of OLE treatment, and subsequently at three month intervals (i.e., week 6, 8, 16, 28, 40 and end of the trial visit at week 52), reflecting a goal of accelerated up-titration, given that all or almost all of the antihypertensive effect occurs within two weeks of treatment (6). If a participant’s recent home mean SBP/DBP values were ≥130 and/or ≥80 mmHg, they were to be up-titrated, barring the absence of a compelling reason not to. The titration was to GMRx2 ½, and if necessary to GMRx2 standard dose, which could be supplemented by the addition of telmisartan 40 mg + amlodipine 5 mg SPC if necessary, and finally spironolactone 25 mg could be added to achieve the desired goal of an average home BP of <130/80 mmHg. Treatment decisions were made by physicians. Study medications were provided free of cost to the participants, and they were instructed to take them once daily. Management of concomitant health conditions was as per usual practice. If participants experienced adverse events considered to be related to the trial medication, down-titration was allowed at the physician’s discretion. At the end of the OLE, the participants were switched to routine usual care.

### Procedures

Participants measured their BP at home, based on the guidance provided by the American Heart Association (7). They used a FORA D40g BP measurement machine (Medisanté BP800; Taidoc Technology Corporation, Taiwan), which is a clinically validated digital cuff-based upper-arm monitor. BP readings were encrypted and automatically sent to the trial database via SIM connection. Measurements were taken over four consecutive days before a trial visit, weekly thereafter, and in triplicate each morning and evening, with morning readings before the next trial treatment dose. Clinic BP was also measured in the seated position at all visits using the same machine model and procedures as mentioned above. Laboratory tests for serum electrolytes and creatinine were performed at week 4, 8, 16 and end of the OLE (week 52). Test for urine albumin-creatinine ratio was performed in participants who had a ≥30% reduction in eGFR and/or a ≥30% increase in serum creatinine between the randomization and week 4 visits during the randomized trial phase, and in all participants at week 52.

### Outcomes

The primary efficacy outcome was percentage of participants with home BP control to <130/80 mmHg at week 52. The primary safety outcome was percentage of participants who discontinued trial treatment due to an adverse event (AE). Secondary outcomes included the percentage of participants with home BP control to <130/80 mmHg and <135/85 mmHg (as recommended by some guidelines), clinic BP control (<140/90 mmHg) at week 52, home and clinic mean BP at week 52, adverse events of special interest (AESIs) (defined as any of the following: symptomatic hypotension, abnormal laboratory findings of sodium, potassium, uric acid, glucose, lipids, creatinine, or estimate glomerular filtration rate (eGFR); headache; peripheral oedema; or any other symptom or laboratory abnormality leading to permanent discontinuation of trial medication), and serious adverse events (SAEs). We also assessed adherence to trial treatment by pill count and therapeutic inertia, defined as non-intensification of BP-lowering therapy despite uncontrolled BP.

### Statistical analysis

The primary efficacy analyses included all participants who were enrolled in the OLE phase, received at least one dose of the trial treatment, and had at least one post-dose home or clinic BP measurement value. The safety population included all participants who were enrolled in the OLE and received at least one dose of the trial treatment. All outcomes were summarized descriptively using means and standard deviations or counts and percentages. Home BP for each participant for week 4, 6, 8, 16, 24, 28, 40, and 52 was calculated as the mean of all BP measurements taken since the clinic visit, prior to these clinic visits.

## Results

### Participant characteristics

All of the 53 participants who completed the randomised phase at the sites where the extension phase was conducted were eligible and invited to participate in the OLE. Of that group, 50 (94%) consented to participate and were enrolled from 21 August 2023, with 48 (96%) completing the extension phase by 20 August 2024. One of the 50 subsequently withdrew consent and another participant discontinued due to personal reasons (Supplementary Figure S2). From the randomized phase placebo, GMRx2 ¼, and GMRx2 ½ arms, 8, 21, and 21 participants, respectively, entered the OLE. Mean age was 49 years, 60% were female, 82% were from Sri Lanka, and 18% from Nigeria. Mean body mass index was 26 kg/m^2^, 26% had dyslipidemia, and 8% had diabetes mellitus (Table 1).

**Table 1:** Summary of characteristics of participants enrolled in open-label extension.


	TREATMENT DURING RANDOMIZED PHASE			

	PLACEBO	GMRx2 ¼	GMRx2 ½	OVERALL

**Participants**	8 (16%)	21 (42%)	21 (42%)	50 (100%)

**Age, years**	47 (15)	48 (8)	50 (7)	49 (9)

**Female**	7 (88%)	13 (62%)	10 (48%)	30 (60%)

**BMI, Kg/m** **^2^**	25.2 (4.2)	28.0 (3.6)	24.8 (3.6)	26.2 (3.9)

***Race***				

**Black or African American**	3 (38%)	2 (10%)	4 (19%)	9 (18%)

**Asian**	5 (62%)	19 (90%)	17 (81%)	41 (82%)

***Country***				

**Sri Lanka**	5 (62%)	19 (90%)	17 (81%)	41 (82%)

**Nigeria**	3 (38%)	2 (10%)	4 (19%)	9 (18%)

***Health conditions***				

**Mild congestive heart failure (NYHA class I or II)**	0 (0%)	1 (5%)	0 (0%)	1 (2%)

**Diabetes mellitus type II**	0 (0%)	4 (19%)	0 (0%)	4 (8%)

**Dyslipidemia**	3 (38%)	5 (24%)	5 (24%)	13 (26%)

**Thyroid disease**	0 (0%)	1 (5%)	1 (5%)	2 (4%)

***Mean (SD) BP at Week 0***				

**Clinic SBP/DBP**	140/84 (8/12)	137/85 (9/9)	135/85 (12/9)	137/85 (10/10)

**Home SBP/DBP**	137/88 (7/10)	136/85 (6/8)	136/84 (6/6)	136/85 (6/7)

***Mean BP (SD) at W*** ***eek 4***				

**Clinic SBP/DBP**	134/83 (11/7)	130/81 (12/8)	131/84 (12/9)	131/83 (12/8)

**Home SBP/DBP**	135/84 (13/7)	124/79 (10/8)	123/77 (11/7)	126/79 (12/8)

**Clinic BP ≥140/90 mmHg at *Week 4***	4 (50%)	2 (10%)	8 (38%)	14 (28%)

**Home BP ≥130/80 mmHg at *Week 4***	7 (88%)	13 (62%)	9 (43%)	29 (58%)


All values are count (percentage) or mean (standard deviation). BMI = body mass index; BP = blood pressure; DBP = diastolic blood pressure; NYHA = New York Heart Association. OLE = Open-label extension; SBP = systolic blood pressure; SD = standard deviation.

### BP levels and BP control rates

Overall home BP control (<130/80 mmHg) was 54% at week 6 and remained ≥53% throughout the follow-up period, reaching 60% at week 52 (Table 2). Similarly, overall home BP control (<135/85 mmHg) rose to 78% at week 6 and remained ≥78% throughout the follow-up period, reaching 90% at week 52. Clinic BP control (<140/90 mmHg) was ≥71% throughout follow-up, reaching 88% at week 52.

**Table 2:** Blood pressure control rates throughout follow-up.


	N	HOME BP <130/80 mmHg	HOME BP <135/85 mmHg	N	CLINIC BP <140/90 mmHg

Week 0	50	2 (4%)	13 (26%)	50	23 (46%)

Week 4	48	21 (44%)	32 (67%)	50	36 (72%)

Week 6	50	27 (54%)	39 (78%)	50	36 (72%)

Week 8	49	26 (53%)	42 (86%)	49	35 (71%)

Week 16	48	27 (56%)	38 (79%)	49	37 (76%)

Week 28	48	33 (69%)	41 (85%)	48	44 (92%)

Week 40	48	33 (73%)	40 (83%)	48	44 (92%)

Week 52	48	29 (60%)	43 (90%)	48	42 (88%)


All values are number of participants (percentage). BP = blood pressure; N = number of participants analyzed.

**Figure 1:**
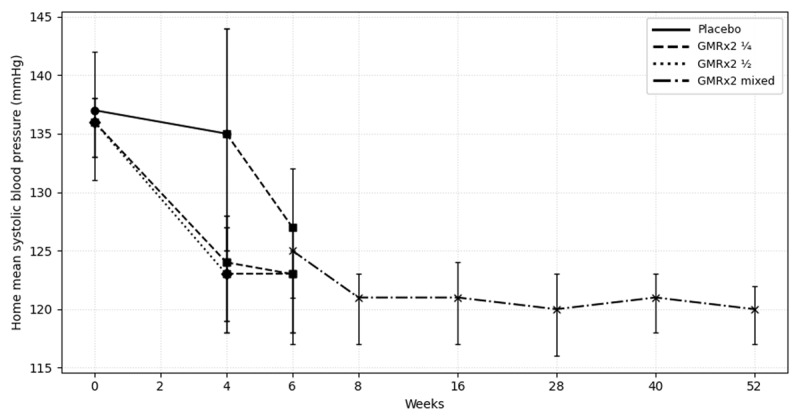
Home systolic blood pressure over time. **Legend:** This figure illustrates, during the randomized double-blind period, from week 0 to 4, participants received placebo, GMRx2 ¼, or GMRx2 ½. At week 4, participants entered the open-label extension phase and were switched to GMRx2 ¼ until week 6. From week 6 onward, at each follow-up visit, if home blood pressure was ≥130/80 mmHg, participants were up-titrated according to the following sequence (GMRx2 mixed): GMRx2 ½ → GMRx2 standard → addition of telmisartan 40 mg + amlodipine 5 mg → addition of spironolactone 25 mg.

Baseline (week 4) mean home and clinic BP levels were 126/79 mmHg and 131/83 mmHg, respectively. Mean home BP was reduced to 121/78 mmHg at week 8 (four weeks into OLE) and was 120/77 at week 52 (Figure 1, Table 3). Similar patterns were seen clinic SBP (Table 3). SBP reductions were observed when participants were switched from placebo to GMRx2 ¼ dose, whereas SBP levels were maintained when switched from GMRx2 groups on to GMRx2 ¼ dose (Figure 1).

**Table 3:** Blood pressure levels throughout follow-up.


		HOME BP MEAN (SD)			CLINIC BP MEAN (SD)	
	
	N	SBP	DBP	N	SBP	DBP

Week 0	50	136 (6)	85 (7)	50	137 (10)	85 (10)

Week 4	48	126 (12)	79 (8)	50	131 (12)	83 (8)

Week 6	50	123 (9)	80 (7)	50	127 (11)	80 (10)

Week 8	49	121 (10)	78 (6)	49	126 (13)	79 (10)

Week 16	48	121 (11)	78 (7)	49	127 (10)	80 (10)

Week 28	48	120 (12)	77 (7)	48	122 (11)	76 (10)

Week 40	48	121 (10)	77 (7)	48	121 (9)	76 (9)

Week 52	48	120 (9)	77 (6)	48	122 (11)	76 (9)


BP = blood pressure; DBP = diastolic blood pressure; N = participants; SBP = systolic blood pressure; SD = standard deviation.

### GMRx2 use pattern, add-on therapy and adherence

At week 52, the proportion of participants receiving GMRx2 ¼, GMRx2 ½, and GMRx2 standard doses was 48%, 24% and 20%, respectively (*Supplementary Figure S3*). Three participants (6%) required add-on therapy with a SPC of telmisartan 40 mg and amlodipine 5 mg in addition to the standard dose of GMRx2—one at week 28 and two at week 40. At week 6, 98% of participants demonstrated ≥80% adherence to the trial treatment, and over 80% maintained ≥80% adherence at any follow-up visit.

### Therapeutic inertia

Twenty-three participants (46%) did not achieve home BP control (<130/80 mmHg) by week 6. Among them, BP-lowering therapy was not intensified in 16 participants, resulting in a therapeutic inertia rate of 70%. Therapeutic inertia for home BP exceeded 70% at weeks 8, 28, and 40 (Supplementary Table 1). Therapeutic inertia for both home and clinic BP was at least 60% at all visits, except for week 16. Consequently, most participants remained on GMRx2 ¼ dose throughout the OLE (Supplementary Figure 3).

### Safety

None of the participants discontinued trial treatment due to an adverse event (Table 4). Thirty (60%) participants experienced at least one AESI, and two (4%) participants had at least one treatment-related adverse event (Table 4). Two (4%) participants experienced at least one serious adverse event, and neither was related to the trial treatment. Twenty-eight (56%) participants had at least one abnormal laboratory finding, most of which related to lipids and sodium, but only three (11%) of them were reported as clinically significant. On average, there was no clinically significant change in any laboratory parameter from baseline to week 52 (Supplementary Table 2).

**Table 4:** Safety Outcomes.


OUTCOME	ALL (N = 50)

From Week 4 to Week 52	

Trial treatment discontinuation due to an adverse event	0 (0%)

Adverse events of special interest	33 (66%)

Symptomatic hypotension	2 (4%)

Abnormal laboratory finding	31 (62%)^1^

Headache	1 (2%)

Peripheral oedema	0 (0%)

Any other symptom or laboratory abnormality that led to permanent discontinuation of trial medication	0 (0%)

Treatment-related adverse events^2^	2 (4%)

Serious adverse event	2 (4%)

Unstable angina	1 (2%)

Acute coronary syndrome	1 (2%)

Acute gastroenteritis	1 (2%)

At Week 52	

Hyponatraemia (sodium <135 mmol/l)	4 (8%)

Hypernatraemia (sodium >145 mmol/l)	5 (10%)

Hypokalaemia (<3.5 mmol/l)	1 (2%)

Hyperkalaemia (>5.5 mmol/l)	0 (0%)


Values are number of participants with ≥1 event and are presented as number of participants (%). ^1^Abnormal lipids (30%), sodium (20%), glucose (18%), uric acid (14%), creatinine (8%), potassium (2%). Total of 11% were reported as clinically significant. ^2^Those reported by the site investigators as possibly, probably or definitely related.

## Discussion

### Summary of key findings

In this open-label extension of a multicentre double-blind randomized trial, a novel low-dose triple combination GMRx2 substantially reduced BP, achieving and maintaining high (60%) home BP control to <130/80 mmHg and high (88%) clinic BP control to <140/90 mmHg, over follow-up despite high (>70%) rates of therapeutic inertia. Most participants received GMRx2 ¼ dose and only three participants were given add-on therapy on top of GMRx2. There was high adherence to therapy and good tolerability, with no participants discontinuing therapy due to adverse events and low rates of hypokalaemia.

### Findings in the context of other relevant evidence

These results should be considered in the context of reports from previous studies of triple combination therapy. In a 36-week, non-comparative, open-label extension of a randomized trial, a triple SPC of olmesartan, amlodipine, and hydrochlorothiazide, prescribed at doses of 20/5/12.5 mg, 40/5/12.5 mg, 40/5/25 mg, 40/10/12.5 mg, or 40/10/25 mg as needed to achieve clinic target BP (<140/90 mmHg or <130/80 mmHg for participants with diabetes, chronic kidney disease, or cardiovascular disease). The triple SPC achieved target BP in 78% of participants, with mean SBP and DBP remaining within 120–140 mmHg and 75–85 mmHg, respectively, throughout the extension phase (8). Several large observational studies conducted in Europe assessed the effects of perindopril/amlodipine/indapamide triple combination. These studies included a total of 22,028 patients evaluating efficacy and tolerability over 3–4 months, with office BP control rates of over 70% (9,10,11). In a non-comparative 16-week study among newly diagnosed adults with hypertension in Rwanda, an SPC of olmesartan, amlodipine, and hydrochlorothiazide prescribed at doses of 10/2.5/6.2 mg, 20/5/12.5 mg or 40/10/12.5 mg achieved a clinic mean BP of 120/76 mmHg and BP control (<140/90 mmHg) in 94% of participants, with high tolerability (12).

In the TRIUMPH open-label randomized trial in Sri Lanka, among adults with uncontrolled hypertension (either untreated or on monotherapy), the use of a low-dose triple SPC—telmisartan 20 mg, amlodipine 2.5 mg, and chlorthalidone 12.5 mg, with the option to up-titrate to double-dose SPC (40/5/25 mg)—significantly improved BP control over six months when compared with usual care (1). However, there was 90% therapeutic inertia rate in the triple SPC group, even higher than that observed in this study, and higher than the 65% rate seen in the usual care group.

Overall, previous studies show that triple SPCs achieve high BP control rates. Addressing therapeutic inertia, which remained high in our study despite the availability of higher doses of GMRx2 and a standard treatment protocol, could yield further benefits.

### Strengths and limitations

This study represents the first long-term, multicentre evaluation of the efficacy and safety of GMRx2, with all relevant efficacy and safety outcomes assessed and reported. Safety evaluations included traditional safety parameters and key biochemical markers relevant to monitoring antihypertensive therapy. From the participating sites, all eligible participants were enrolled except one, and all but two participants completed the study, minimising the potential for selection and attrition bias. However, some limitations should be acknowledged. The sample size was small, and participants were exclusively from Sri Lanka and Nigeria, which may limit the generalisability of the findings. Additionally, the interventions and outcome assessments were not blinded. While this could introduce bias, it is unlikely to have significantly affected BP measurements, as digital BP monitors were used, and values were automatically transferred to trial database via sim connection. Similarly, the unblinded assessment of safety outcomes could introduce bias against the new intervention; however, this risk may have been mitigated by focusing on adverse events of special interest and relying on objective laboratory parameters for safety evaluations.

### Implication for clinical practice and research

Patient adherence to treatment and therapeutic inertia are significant barriers to achieving optimal BP control. Adherence to BP-lowering therapy in the extension phase remained high, indicating good patient acceptability and highlighting the potential convenience and efficacy of a once-daily, single-pill regimen for long term BP reduction and control. However, despite recommendations to site staff to adhere to treatment protocols and titrate therapy in cases of uncontrolled BP (unless contraindicated), therapeutic inertia remained high despite the simplicity of intensifying therapy. This highlights a critical challenge in implementing evidence-based interventions, and it is also a realistic reflection of implementation challenges in routine clinical practice. The exact reasons for therapeutic inertia in this context need to be explored. Achieving optimal BP control is unlikely without effectively addressing therapeutic inertia in clinical practice. Future research should focus on understanding provider-related reasons for therapeutic inertia and developing strategies to address them.

Additionally, broader studies across diverse populations and healthcare systems will be required to confirm the generalisability of our findings, and these studies should also evaluate the cost-effectiveness of GMRx2-based treatment for hypertension.

While initiation of antihypertensive therapy with dual combination ideally as a SPC is already established and recommended by contemporary international guidelines, initiation with a low-dose triple SPC appears to result in even better BP control and therefore improve outcomes.

## Conclusion

GMRx2, as a novel low-dose triple SPC, offers an effective, durable, and tolerable option for BP reduction. Further improvements in BP reduction and control can be achieved with GMRx2 by addressing therapeutic inertia. With treatment options like GMRx2, achieving BP control in more than 80% of treated patients is Feasible.

## Additional File

The additional file for this article can be found as follows:

10.5334/gh.1481.s1 Supplementary files. Supplementary Tables 1 to 2, and Figures S1 to S3.
