# Supplementary material for: Long-Term Efficacy and Safety of a Novel Low-Dose Triple Single-Pill Combination for the Treatment of Hypertension
Source: Glob Heart. 2025 Oct 31;20(1):102. doi: 10.5334/gh.1481 (PMC12577540; doi:10.5334/gh.1481)
Supplement: Supplementary files. — Supplementary Tables 1 to 2, and Figures S1 to S3. [file gh-20-1-1481-s1.pdf]

Supplementary file

***Supplementary Figure S1*: Study Schema**

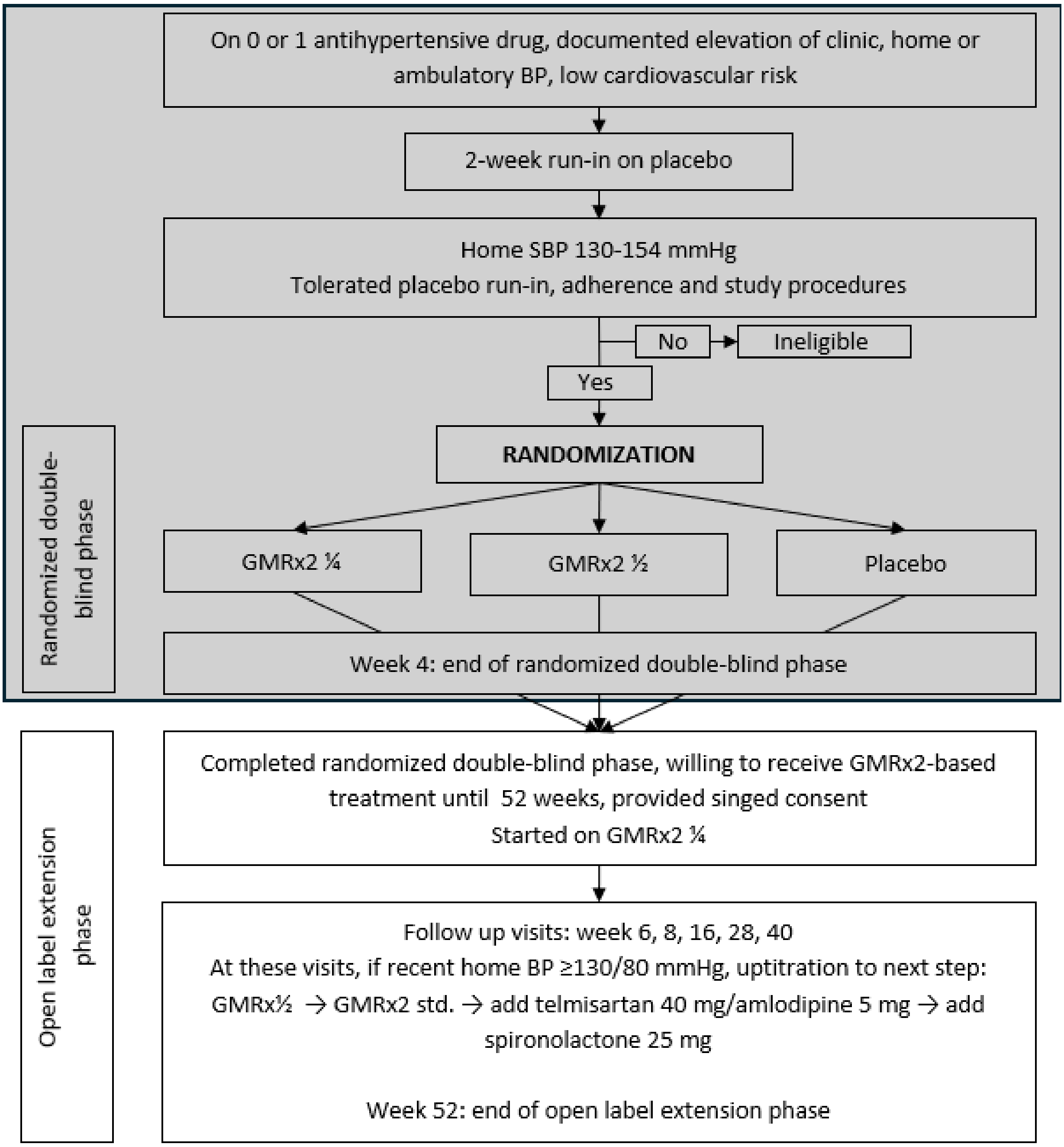

Supplementary Figure S2: CONSORT chart for participant enrolment and follow up

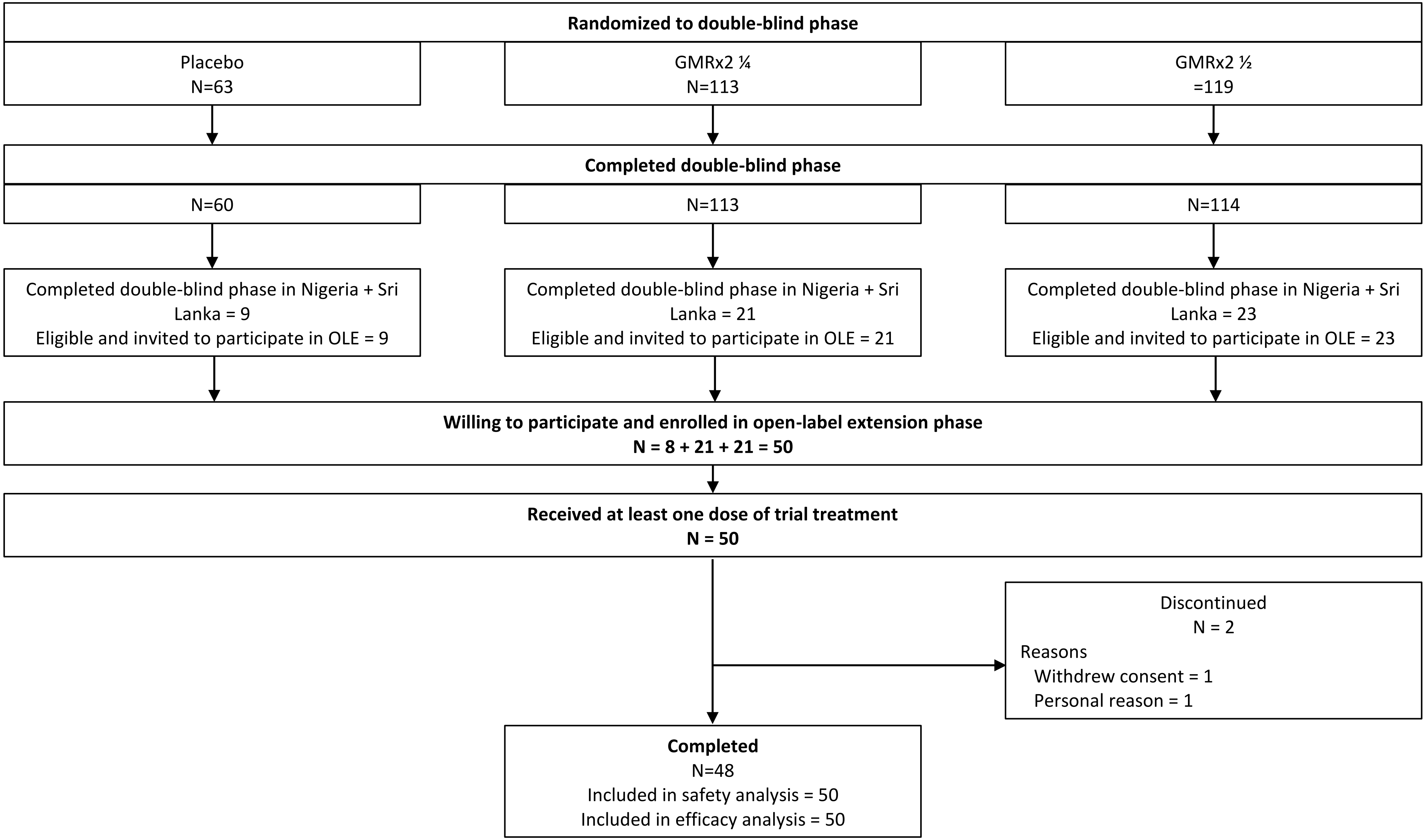

Supplementary Figure S3: Antihypertensive drug regimens during the randomized and open-label extension phase

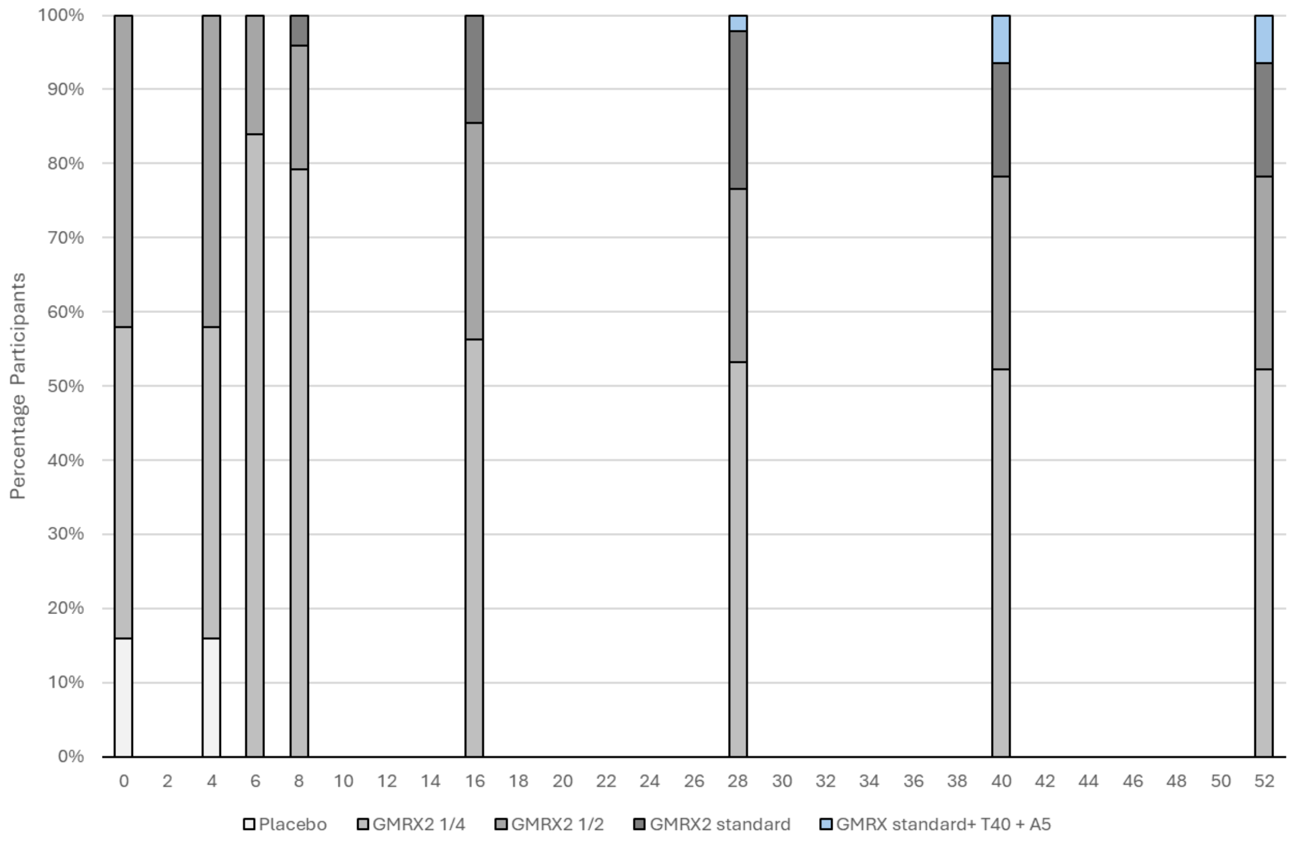

Supplementary Table S1: Therapeutic inertia by home and clinic blood pressure

	Home BP[1]		Clinic BP		Home and clinic BP	
	Uncontrolled home BP[2]	Therapeutic inertia[3]	Uncontrolled clinic BP[4]	Therapeutic inertia[3]	Uncontrolled home and clinic BP[5]	Therapeutic inertia[3]
Week 6	23 (46%)	16 (70%)	14 (28%)	11 (79%)	5 (10%)	3 (60%)
Week 8	23 (47%)	17 (74%)	14 (29%)	12 (86%)	8 (16%)	6 (75%)
Week 16	21 (44%)	7 (33%)	12 (24%)	5 (42%)	8 (17%)	2 (25%)
Week 28	15 (31%)	11 (73%)	4 (8%)	4 (100%)	1 (2%)	1 (100%)
Week 40	13 (27%)	11 (85%)	4 (8%)	3 (75%)	3 (6%)	2 (67%)
Week 52	19 (40%)	Not applicable	6 (13%)	Not applicable	4 (8%)	Not applicable

All values are number and percentage of participants
[1] Defined as BP before a visit and after the previous visit
[2] ≥130/80 mmHg
[3]Antihypertensive drug therapy not intensified despite uncontrolled BP
[4] ≥140/90 mmHg
[5] Home BP ≥130/80 mmHg and clinic BP ≥140/90 mmHg
BP = blood pressure

Supplementary Table S2: Laboratory parameters

	Sodium, mmol/L	Potassium, mmol/L	Chloride, mmol / L	Uric acid, mmol/L	Creatinine, mg/dl	Participants with eGFR <60 mL/min/1.73 m²	Albumin-Creatinine ratio, mg/g	Fasting blood glucose, mmol/L	Total cholesterol, mmol/L	Low-density lipoprotein cholesterol, mmol/L	High-density lipoprotein cholesterol, mmol/L
	Mean (SD)	Mean (SD)	Mean (SD)	Mean (SD)	Mean (SD)	N (%)	Mean (SD)	Mean (SD)	Mean (SD)	Mean (SD)	Mean (SD)
Week 0	142.1 (3.8)	4.3 (0.4)	104.0 (2.5)	0.3 (0.1)	0.7 (0.2)	0 (0%)	31.8 (119.1)	5.4 (1.0)	5.3 (1.3)	3.4 (1.2)	1.3 (0.3)
Week 4	141.5 (3.8)	4.3 (0.4)	103.0 (2.9)	0.3 (0.1	0.8 (0.2)	0 (0%)	29.0 (81.1)	5.5 (1.5)	5.1 (1.0)	3.3 (01.0)	1.2 (0.3)
Week 8	140.4 (3.5)	4.2 (0.4)	102.2 (2.6)		0.8 (0.2)	0 (0%)					
Week 16	139.8 (3.4)	4.2 (0.4)	101.8 (3.3)		0.8 (0.2)	0 (0%)					
Week 52	139.6 (3.9)	4.0 (0.4)	102.7 (3.8)	0.3 (0.1)	0.8 (0.2)	0 (0%)	32.5 (85.4)	5.3 (0.8)	5.3 (1.2)	3.4 (1.1)	1.4 (0.7)

Weeks represent weeks since randomization in the double-blind phase

Site Investigators and staff

Sri Lanka
A de Silva, Colombo North Teaching Hospital
N Fernando, National Hospital of Sri Lanka
G Galappatthy, National Hospital of Sri Lanka
P Lakshman, Jaffna Teaching Hospital
G Mayurathan, Kandy National Hospital
T Pereira, Colombo South Teaching Hospital
M Rahuman, National Hospital of Sri Lanka
G Ranasinghe, National Hospital of Sri Lanka
L Rasnayake, Kurunegala Teaching Hospital
W Uluwattage, Karapitiya Teaching Hospital

Nigeria
Dike Ojji, University of Abuja, Nigeria
Nanna Ripiye, University of Abuja, Nigeria
Patrick Ponzing, University of Abuja, Nigeria
Michael Ochem, University of Abuja, Nigeria

Mahmoud Umar Sani, Aminu Kano Teaching Hospital, Nigeria
Shehu A. Kana, Aminu Kano Teaching Hospital, Nigeria
Abdulgafar Lekan Olawumi, Aminu Kano Teaching Hospital, Nigeria
Tijjani Abdussalam, Aminu Kano Teaching Hospital, Nigeria
Zuwaira Maaruf, Aminu Kano Teaching Hospital, Nigeria
Kamal Ibrahim, Aminu Kano Teaching Hospital, Nigeria

Steering committee

Member	Affiliation
Professor Paul Whelton (Chair)	Tulane University School of Public Health and Tropical Medicine, New Orleans, USA
Professor William C Cushman	University of Tennessee Health Science Center, Memphis, Tennessee, USA
Professor Asita de Silva	University of Kelaniya, Sri Lanka
Professor Gian Luca Di Tanna	University of Applied Sciences and Arts of Southern Switzerland, Switzerland
Professor Diederick Grobbee	University Medical Center Utrecht, Utrecht University, The Netherlands
Professor Krzysztof Narkiewicz	Medical University of Gdańsk, Poland
A/Professor Dike Ojji	University of Abuja, Nigeria
Professor Suzanne Oparil	University of Alabama at Birmingham, USA
Professor Neil Poulter	Imperial College, London, UK
Professor Anthony Rodgers	The George Institute for Global Health, University of New South Wales, Australia
Dr Abdul Salam	The George Institute for Global Health, University of New South Wales, India
Professor Markus P Schlaich	The University of Western Australia, Australia
Professor Aletta E Schutte	The George Institute for Global Health, University of New South Wales, Australia
A/Professor Wilko Spiering	University Medical Center Utrecht, Utrecht University, The Netherlands
Professor Bryan Williams	University College London, UK
Professor Jackson T Wright Jr	University Hospitals Cleveland Medical Center, Case Western Reserve University, USA

Independent Data Monitoring Committee

Member	Affiliation
Professor Lawrence Appel (Chair)	Johns Hopkins University, Baltimore, USA
Professor Mark Espeland	Wake Forest University, Winston Salem, USA
Professor Michael Weber	SUNY Downstate Medical Centre, New York, USA

Endpoint Adjudication Committee

Member	Affiliation
Professor James L Januzzi (Chair)	Harvard Medical School, Boston, USA
Professor Gregory Fulcher	University of Sydney, Sydney, Australia
Professor John Watson	Australian National University, Canberra, Australia
Professor Scott Kasner	University of Pennsylvania, Pennsylvania, USA

Trial organization

Sponsor co-ordination – Kevin Spivey, Karl Roberts, Rene Gonzalez
International and regional co-ordination, George Clinical Pty Ltd - Imran Mubashir, Gopal Pai, Alina Yoffe, Dulesh Peiris, Masego Johnstone, Shailesh Hegde, Suzanne Milne, Alison Richardson, Andrejs Faibusevics, Christine Adeyeri, Nicole Khamo, Chanel Kuffour, Murali Dhakshinamurthy, Libin Zhang, Caren Andexer, Haley Austill, Rajesh Kumar Karthikeyan, Tarun Parashar, Prakash Murugeshan, Amrutha Nagarajaiah, Chaitra Hindiganad, Mahesh Basavaraj, Sneha Rao, Vipin Jose
Regional Co-ordination, Imperial Clinical Trials Unit - Gaia Kiru, Claire Brennan, Jennifer Murphy, Lisa Feng, Felicia Frost, Irene Aboh, Egiehiokhin Obanor, Marta Szajna, Rosemary Keshinro, Margarita Durkina
Regional Co-ordination, Remedium One – Poornima Ellawala, Anuradha Dahanayake, Niran Perera
Academic Co-ordinating Centre, The George Institute for Global Health – Ruth Freed, Rachel Harris
Statistical consulting, Veristat Plc: Ralf Wu, Christian Graven Robin Bliss, Sarah Mulholland, Wendy Pan, Joel Clarke, Nancy Hsieh

Contributors

AS wrote the first draft with input from AR. Statistical analyses were conducted by RP. The Steering Committee had full access to all data. All authors commented on draft manuscript and supported the decision to submit.

Acknowledgements

Pharmalex/Cencora: Padma Uma Devi (proof-reading)
George Medicines: Amy K. Carroll (proof-reading)

Data sharing statement

Documentation around the analyses presented here will be made available to qualified scientific and medical researchers, upon researcher's request, as necessary for conducting legitimate research. Patient data will be de-identified and will be shared via a secure means. Requests for data will only be reviewed after approval of the product in the USA and EU, and after George Medicines approval of its Data Access Request and receipt of its executed Data Sharing Agreement. A request form can be obtained by email to the corresponding author or to info@george-medicines.com
